# Lifestyle index for mortality prediction using multiple ageing cohorts in the USA, UK and Europe

**DOI:** 10.1038/s41598-018-24778-1

**Published:** 2018-04-27

**Authors:** Jing Liao, Graciela Muniz-Terrera, Shaun Scholes, Yuantao Hao, Yu-ming Chen

**Affiliations:** 1 Department of Medical Statistics and Epidemiology, School of Public Health, Sun Yat-sen University, No.74 Zhongshan 2nd Road, Guangzhou, 510080 P.R. China; 2 Sun Yat-sen Global Health Institute, Institute of State Governance, Sun Yat-sen University, No. 135 Xingang West Road, Guangzhou, 510275 P.R. China; 3 Biostatistics and Epidemiology, Centre for Dementia Prevention, University of Edinburgh, Edinburgh, Scotland; 4 UCL Research Department of Epidemiology and Public Health, Faculty of Population Health Sciences, University College London, 1-19 Torrington Place, London, WC1E 6BT UK

## Abstract

Current mortality prediction indexes are mainly based on functional morbidity and comorbidity, with limited information for risk prevention. This study aimed to develop and validate a modifiable lifestyle-based mortality predication index for older adults. Data from 51,688 participants (56% women) aged ≥50 years in 2002 Health and Retirement Study, 2002 English Longitudinal Study of Ageing and 2004 Survey of Health Ageing and Retirement in Europe were used to estimate coefficients of the index with cohort-stratified Cox regression. Models were validated across studies and compared to the Lee index (having comorbid and morbidity predictors). Over an average of 11-year follow-up, 10,240 participants died. The lifestyle index includes smoking, drinking, exercising, sleep quality, BMI, sex and age; showing adequate model performance in internal validation (C-statistic 0.79; D-statistic 1.94; calibration slope 1.13) and in all combinations of internal-external cross-validation. It outperformed Lee index (e.g. differences in C-statistic = 0.01, D-statistic = 0.17, P < 0.001) consistently across health status. The lifestyle index stratified participants into varying mortality risk groups, with those in the top quintile having 13.5% excess absolute mortality risk over 10 years than those in the bottom 50th centile. Our lifestyle index with easy-assessable behavioural factors and improved generalizability may maximize its usability for personalized risk management.

## Introduction

Lifestyle factors contribute substantially to chronic diseases and mortality even in old age^1^, with unhealthy behaviours tending to occur together and exert a synergistic effect on health^2^. To investigate the combined effects of multiple behavioural factors on health, most epidemiological studies have generated a lifestyle index by adding the number of dichotomous risky or non-risky behavioural indicators^1,3,4^. This approach is straightforward but may arbitrarily assign behaviours into the ‘risky’ category and overlook the dependence between behaviours and their relative importance to health^3^.

On the other hand, risk indices derived from prediction models can generate more objective estimations of multiple risk factors on health and further predict the absolute risk of an event for individuals with certain risk profiles over a specific time period^5^. So far, however, few mortality prediction models for community-dwelling older adults have been constructed. Existing indices focus mainly on functional morbidity measures^6,7^ or involve 11–12 risk factors^8,9^ including demographic characteristics and self-reported comorbid and functional conditions to predict up to 10-year mortality^10,11^. Their predictive value notwithstanding, these indices cannot provide information for risk intervention. The prevention of disease development and functional decline is better achieved through upstream modifiable lifestyle factors^12^. Additionally, most indices were derived in a single sample of North American older adults and lack external validation^13^.

Therefore, this study aimed to develop and validate a mortality prediction index based on modifiable lifestyle factors using three population-representative ageing cohorts in the US, UK and Europe. These internationally collaborative epidemiologic cohorts enable detailed investigation on the predictive performance of our lifestyle index, while taking between-cohort heterogeneity into account^14,15^. A lifestyle index for mortality that performs well across cohorts enhances its credibility and generalizability, and may then be utilized for risk prevention through behaviour changes.

## Results

### Characteristics of the Study Sample

Among the 51,688 participants, 10,240 deaths (19.8%) occurred over an average of 11 years of follow-up (dates varied, HRS 13 years, ELSA 11 years and SHARE 9.5 years). The distribution of baseline lifestyle measures varied between cohorts (Supplementary Table S1) that higher percentages of SHARE participants were never smokers, heavy drinkers and were physically active; whereas ELSA participants were moderate drinkers, slept restlessly and were overweight or obese.

### Lifestyle-Based Risk Prediction Model for Mortality

The cohort-stratified Cox regression of multiple lifestyle factors for mortality indicated that smoking, physically inactivity and underweight were associated with increased mortality, while restless sleep was an independent risk factor for men only (Table 1). Regression estimates of lifestyle factors for mortality were similar across cohorts (results not shown), except that heavy drinking was only a risk factor in HRS (p for cohort interaction <0.01). Conditional on risk predictors included, the three cohorts showed similar baseline distributions which can be jointly modelled as a restricted regression spline with 1 internal knot (Supplementary Fig. S1).

**Table 1:** Baseline lifestyle measures and associations with mortality cases in 2013–2014 (N = 51,688).

Predictors	Mean or %	β^a^ [95%CI]
**Ln-age**	4.2	6.343 [5.737,6.949]
**Gender**		
Male	44.4	Ref.
Female	55.6	−0.476 [−0.574,−0.377]
Female*Lnage		0.589 [0.092,1.086]
**Smoking status**		
Never smoker	51.0	Ref.
Ex-smoker	32.4	0.330 [0.262,0.399]
Current smoker	16.6	0.771 [0.672,0.871]
**Drinking status**		
Nondrinker	38.3	0.345 [0.270,0.421]
Former drinker	10.8	0.131 [0.027,0.234]
Moderate drinker	37.9	Ref.
Heavy drinker	13.0	0.106 [−0.025,0.237]
**Physical activity level**		
High	40.1	−0.417 [−0.496,−0.338]
Medium	44.5	Ref.
Low	15.4	0.770 [0.683,0.856]
**Sleep restless**		
No	68.6	Ref.
Yes	31.4	0.211 [0.118,0.304]
Female*Yes		−0.260 [−0.396,−0.125]
**BMI**		
≤20	4.3	0.442 [0.308,0.577]
>20–25	34.7	0.160 [0.091,0.229]
>25–30	41.1	Ref.
>30	19.9	0.115 [0.036,0.195]

Data: Health and Retirement Study, English Longitudinal Study of Ageing and Survey of Health, Ageing and Retirement in Europe.

BMI, Body mass index; CI, confidence interval; Ref., reference group.

^a^Beta coefficients were derived from Cox regression models stratified by cohort. Statistically-significant sex interaction terms were in italic with * signs. Age was naturally logarithmically transformed to improve the model fit and was centred at 66 years.

### Model Validation

The lifestyle prediction model showed good discrimination (Table 2). The pooled C-statistic and D-statistic estimates of internal validation were 0.79 and 1.94 with similar performance in each round of IECV (i.e., C-statistic range 0.77–0.81, D-statistic range 1.85–2.06), indicating good discrimination consistently across cohorts. Regarding calibration, the pooled calibration slope 1.13 was slightly larger than 1.0, suggesting that the predicted mortality risk may be lower than the observed risk. Comparable findings were drawn from IECV analysis of the calibration slope and calibration plots (Supplementary Fig. S2), showing that the under-prediction of mortality risk was most evident in ELSA. Our lifestyle-based model outperformed Lee’s model^8,10^ that includes comorbid conditions (i.e., diabetes mellitus, cancer, lung disease and heart failure) and functional variables (i.e., bathing, managing finances, walking several blocks and pushing/pulling heavy objects), showing consistently improved discrimination, e.g. the difference between two indexes was 0.01 for C-statistic (P < 0.001) and 0.17 for D-statistic (P < 0.001), and calibration (1.13 vs 0.80) in the total subsample, as well as among participants with and without clinical conditions (Table 2). This subgroup analysis further indicated that our lifestyle index seemed to perform better in healthy participants than those with clinical conditions.

**Table 2:** Lifestyle-based predication model performance.

Models	N	C-statistic β [95%CI]		D-statistic β [95%CI]		Calibration slope β [95%CI]	
**Internal validation** ^a^	51,688	0.792	[0.787,0.796]	1.937	[1.901,1.972]	1.125	[1.087,1.162]
**Internal-external cross-validation** ^b^							
HRS & ELSA (D)	25,390	0.788	[0.783,0.793]	1.979	[1.912,2.048]	0.964	[0.936,0.992]
SHARE (V)	26,298	0.805	[0.797,0.813]	2.058	[1.951,2.165]	0.965	[0.919,1.010]
HRS & SHARE (D)	41,177	0.798	[0.793,0.803]	2.068	[2.010,2.126]	1.073	[1.035,1.111]
ELSA (V)	10,511	0.803	[0.794,0.812]	1.875	[1.791,1.958]	1.045	[0.985,1.105]
ELSA & SHARE (D)	36,809	0.804	[0.798,0.811]	2.064	[1.963,2.165]	1.030	[0.990,1.071]
HRS (V)	14,879	0.773	[0.767,0.779]	1.846	[1.779,1.912]	0.996	[0.958,1.033]
**Lifestyle index vs. Lee index** ^c^							
**Lifestyle index**							
Total	29,105	0.764	[0.758,0.769]	1.738	[1.676,1.800]	1.130	[1.086,1.174]
With clinical conditions	11,716	0.729	[0.721,0.736]	1.445	[1.365,1.525]	0.972	[0.917,1.026]
Without clinical conditions	17,389	0.771	[0.763,0.780]	1.877	[1.781,1.972]	1.183	[1.115,1.252]
**Lee index**							
Total	29,105	0.751	[0.745,0.756]	1.564	[1.492,1.636]	0.798	[0.766,0.830]
With clinical conditions	11,716	0.712	[0.704,0.719]	1.276	[1.188,1.364]	0.751	[0.706,0.795]
Without clinical conditions	17,389	0.744	[0.736,0.753]	1.695	[1.598,1.791]	1.047	[0.984,1.110]

Data: Health and Retirement Study, English Longitudinal Study of Ageing and Survey of Health, Ageing and Retirement in Europe.

HRS, Health and Retirement Study; ELSA, English Longitudinal Study of Ageing; SHARE, Survey of Health, Ageing and Retirement in Europe; CI, confidence interval.

^a^Internal validation was assessed using a weighted meta-analysis that the pooled estimates were the weighted averages of study-specific estimates.

^b^Internal-external cross-validation interactively withheld one of the cohort (V: validation cohort) to externally validate the prediction model derived from the other remaining cohorts (D: development cohorts).

^c^Comparison was conducted in a subsample that had all measures of both lifestyle index and Lee index (Lee *et al*., 2006.), and separately for these with/without clinical conditions (i.e., diabetes mellitus, cancer, lung disease and heart failure at baseline).

### Lifestyle Index for Mortality

**Figure 1:**
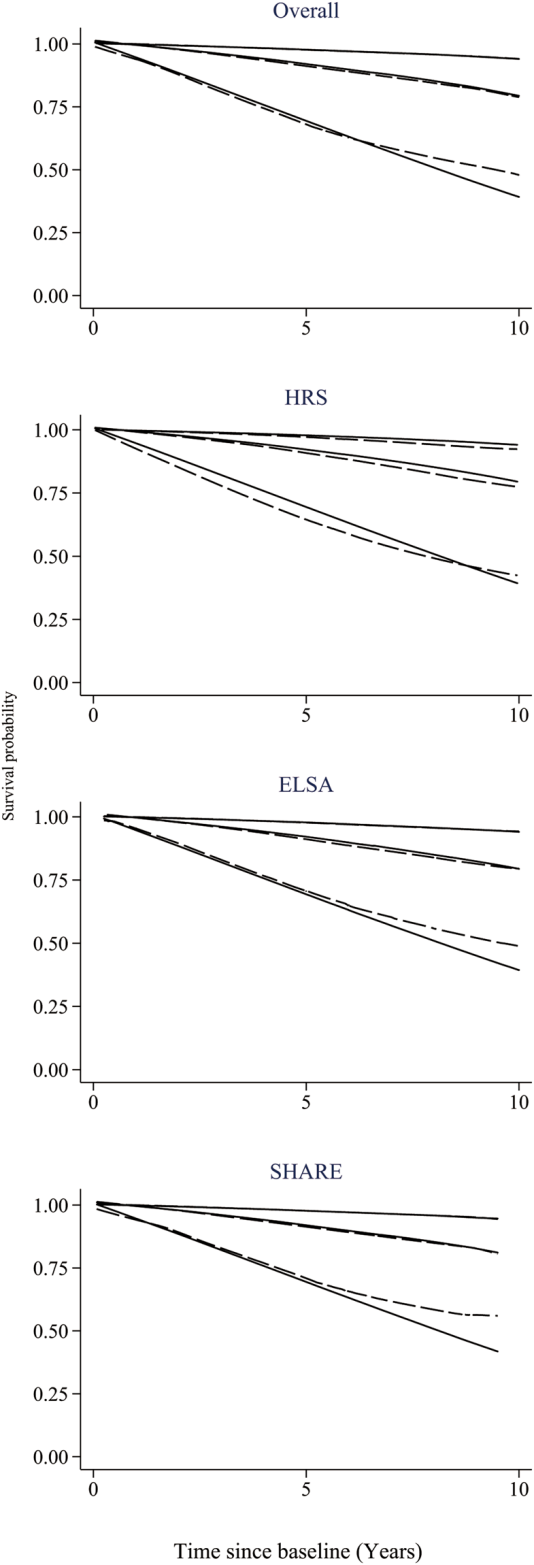
Kaplan-Meier survival curves (solid lines) and estimated average survival curves (dash lines) for three prognostic groups in the pooled data and in each cohort. The groups in each plot are defined by the cut-points at 50^th^ and 80^th^ centiles of the lifestyle index. HRS: Health and Retirement Study; ELSA, English Longitudinal Study of Ageing; SHARE: Survey of Health, Ageing and Retirement in Europe.

Table 3 shows the lifestyle-based index that consists of points for each risk predictor, total points and corresponding absolute risk of 10-year mortality. In our study sample, the lifestyle index ranged from −2 to 15 (mean [SD], 4.3[3.0]), which effectively stratified individuals into groups with varying mortality risks. Kaplan-Meier survival curves for three prognostic groups at 50^th^ and 80^th^ centiles of the lifestyle index (i.e., 4 and 7 points) showed reasonably good agreement with the estimated survival curves, and the separation between groups was well maintained across the individual cohorts (Fig. 1).

**Table 3:** Lifestyle-based index for10-year mortality risk in older adults.

Points		−1	0	1	2	3	4	5	6	7	8			
Age			50–54	55–59		60–64	65–69	70–74	75–79	80–84	≥85			
Sex		Female	Male											
Smoking status			Never	Past	Now									
Drinking status			Former/Moderate	None/Heavy										
Physical activity level		High	Medium		Low									
Restless sleep			No	Yes (Male)										
BMI (weight/height^2^)			>20	≤20										
**Total Points (TP)**	−**2**	−**1**	**0**	**1**	**2**	**3**	**4**	**5**	**6**	**7**	**8**	**9**	**10**	**11+**
**10-y mortality risk%**	0.4	0.6	0.9	1.3	2.1	3.1	4.8	7.3	10.9	16.3	23.9	34.2	47.5	62.8
**Risk groups**	50^th^ centile						50^th^-<80^th^ centile			≥80th centile				

Data: Health and Retirement Study, English Longitudinal Study of Ageing and Survey of Health, Ageing and Retirement in Europe^a^.

^a^A summary risk score for each participant was calculated as the total points for all risk predictors present. For example, a participant aged 60–64 (score 3), male (0), being a current smoker (2), low in physical activity (2), restless sleep (1), and BMI = 20 (1) has total points of 9, and associated with a 34.2% absolute mortality risk in 10 years.

## Discussion

We developed and validated a lifestyle-based risk index for mortality of older adults aged 50 years and above, using three comparable ageing cohorts in the US (HRS), UK (ELSA) and Europe (SHARE). The index includes modifiable smoking, drinking, exercising, sleeping quality and BMI, besides age and sex. It validated rather well in terms of discrimination, but less well regarding calibration to the extent that the mortality risks may be underestimated in high-risk individuals. Nonetheless, our lifestyle index effectively stratified older adults into varying mortality risk groups and demonstrated equivalently adequate performance in each round of internal-external cross-validation. It is thus reasonable to present a single lifestyle risk index with improved generalizability based on data from all three cohorts.

Our lifestyle index identifies high risk older adults via the most common and easily-assessable lifestyle factors, and showed better performance than indices with comorbidities and functional status^8,10^ regardless of participants’ health status. The development of clinical conditions may be on the pathway of lifestyle factors to mortality, as such weakening the predictive value of lifestyle risk factors^12^. Prediction models based on upstream lifestyle risk factors are of the most relevance for disease prevention, and provide useful information for personalized intervention targeted on modifiable unhealthy behaviours. Our lifestyle index with adequate performance in the absence of clinical risk factors enables itself to be easily implemented into different care settings.

Moreover, our lifestyle index for mortality exceeds previous indices by demonstrating its generalizability across three independent ageing cohorts. Current mortality prediction indices for older adults were predominately developed in a single homogenous sample of the American population^6–9^, which cannot be directly transported to other populations. Our study instead derived the lifestyle index from diverse populations representing several countries, carefully accounting for the between-cohort heterogeneity^16,17^. We fitted one-stage stratified models that generate similar risk scores over multiple cohorts as a two-stage fixed- or random-effects meta-analysis, and has the advantage of simplicity^14,15^. We found comparable proportional associations of lifestyle predictors on mortality across the selected cohorts, except for a weak heterogeneity in the heavy drinking effect between HRS and SHARE male participants only. We also found similar baseline distributions among three cohorts conditional on lifestyle factors included, which validated the estimation of a pooled baseline function^18^. Our findings were in line with results from other multi-country and cohort pooling studies^19,20^, which may imply similar underlying biology of lifestyle factors across these demographics.

External validation is essential for a prognostic model’s clinical and public health applications^13,21^. Only one of the current mortality indices for older adults was tested in an independent sample of new respondents to the original survey^11^, but under the identical survey setting the variation between the development and validation datasets was minor. We employed the IECV approach as suggested by recent literature reviews^14,16^ to evaluate the lifestyle index’s performance regarding both discrimination and calibration. For all combinations of the IECV, good discrimination was indicated by both C-statistic and D-statistic concordantly, as well as fairly adequate calibration in the face of some evidence of underprediction in the high-risk groups (<20% of the study sample). Given discrimination is a more relevant indicator for risk stratification than calibration^21^, our lifestyle-based index performed consistently well across all cohorts.

Our study developed a robust lifestyle-based prognostic index that validated across three large population-representative ageing cohorts. This index has the advantages of simplicity, increased precision and generalizability, which enable better transportability than prognostic models derived from a single dataset. However, several limitations should be noted. First, despite efforts taken to harmonizing these datasets, our study may still be affected by variations in lifestyle measures. Thus, the apparent differences in baseline lifestyle distribution between cohorts may reflect a combination of variations in survey questions, social- and cultural-biases in self-reported measures, and true between-cohort differences. Also, confined to lifestyle measures available in these surveys, our index was unable to include all lifestyle risk factors for mortality. This parsimonious index yet covers these common and critical lifestyle factors related to mortality^1^ and provides better stability and transportability across multiple studies than comprehensive indices^13,18^. Second, we focused on lifestyle measures 10 years before death events to minimize the reversed influence from poor health to behaviours. However, this one-time assessment cannot take into account changes in lifestyle patterns over time, and could lead to an underestimation of the average cumulative exposure to these factors on mortality^22,23^. Although within-person change in health-related behaviours may not be substantial in older adulthood^22^, incorporating behavioural patterns of multiple waves would nevertheless improve the precision of the current analysis. Third, SHARE covers most of the European Union countries with inevitably different lifestyle characteristics and health status. Nevertheless, we did not find statistically-significant differences in lifestyle-mortality associations among these countries (result not shown), and hence used them collectively as representing the average level of Europe. Fourth, we adopted the IECV approach to maximize the number of datasets available for model development as well as for external validation, but we note that this approach is only partially external^18^ and may be limited given the number of selected cohorts in our study is small^16,17^. Last, as the present study was mainly focused on North American and European Caucasian older adults, its generalizability to other non-white populations requires further external validation.

In conclusion, this study developed a lifestyle-based index to predict 10-year mortality risk among community-dwelling older adults of the US, UK and Europe. In terms of validation, the lifestyle index showed consistently adequate discrimination and calibration across these geographically and socio-demographically diverse cohorts. Our lifestyle index demonstrated simplicity, credibility and generalizability and may serve as a practical tool for personalized risk assessment and management in older adults.

## Methods

### Study Sample

Data from three comparable national panel surveys were used, namely the Health and Retirement Study (HRS)^24^, the English Longitudinal Study of Ageing (ELSA)^25^ and the Survey of Health Ageing and Retirement in Europe (SHARE)^26^. All the procedures were conducted in accordance with the approved guidelines and informed consent was obtained from all participants of respective cohort. HRS was approved by the University of Michigan Health Science/Behavioural Sciences Institutional Review Board, ELSA was approved by the London Multicentre Research Ethics Committee, and SHARE was approved by the Ethics Committee of the University of Mannheim and the Ethics Council of the Max Planck Society.

The present study included participants who were 50 years and older in the 2002 HRS, the 2002 ELSA, and the 2004 SHARE, and had complete lifestyle measures and linkage to death records up to 2013–2014. This resulted in an analytical sample of 51,688 (56% women). Participants with missing measures (11% HRS, 8% ELSA, 2% SHARE) tended to be older, male and less educated than the study sample.

### Mortality Ascertainment

All surveys trace participants’ mortality status and conduct exit interviews with the decedent’s spouse, child or other informant. We used mortality data updated to the end of 2013 (2014 for HRS). HRS and ELSA also monitor vital status by locating respondents with the National Death Index or the National Health Service’s Central Registry.

### Risk Predictors

Besides age and sex, we included well-established lifestyle factors of mortality in accordance with the literature^1,2^. Full details on the harmonization of the baseline measures across the studies is provided in supplementary text (Supplementary Text S1). Smoking status was categorized as non-smoker (reference), ex-smoker or current smoker. Drinking status was coded as non-drinker (zero-alcohol intake), former drinker, moderate (≤two drinks/day, reference) and heavy drinker (>two drinks/day). Physical activity level was defined as high (vigorous exercise at least once per week), medium (moderate exercise at least once per week, reference) and low (hardly ever/never moderate-vigorous exercise). Sleep quality was measured by ‘whether sleep was restless’ (No = 0, Yes = 1). Body mass index (BMI, kg/m^2^) was grouped into four classes ≤20, >20–25, >25–30 (reference) and >30 kg/m^2^.

### Statistical analysis

We used Cox regression stratified by cohort to estimate coefficients of the lifestyle model. Risk predictors were modifiable health-related behaviours namely smoking, drinking, physical activity, sleep quality and BMI, as well as sex and age (a natural log transformation was used to improve model fit). Backward selection was used, and variables that were independent predictors of mortality in the multivariable models (p < 0.05) were included in the final prediction model. We tested sex variations in all associations by using interaction terms, and examined the between-cohort heterogeneity in predictor-mortality associations and in baseline distribution functions; whereby a pooled baseline survival function was estimated via flexible parametric proportional hazards models^18^ using a restricted natural cubic spline function of log time.

We validated the lifestyle prediction model by assessing discrimination and calibration. Discrimination (i.e. the ability of a predictive model to separate those who died from those who did not) was assessed by Harrell’s Concordance (C-statistic)^27^ and Royston and Sauerbrei’s separation Discrimination (D-statistic)^28^. Larger values indicate better discrimination. Calibration (which reflects prediction accuracy) was evaluated by the calibration slope and calibration plot. A calibration slope closes to 1.0 suggests good calibration, while the calibration curve going through the origin with a 45-degree slope indicates perfect prediction. We validated the prediction model in three ways. First, we conducted internal validation across cohorts using a weighted meta-analysis, where estimates from individual cohorts were combined to obtain the weighted average^15^. Second, we adopted the internal-external cross-validation (IECV) approach^18^ that interactively withheld data from one of the cohort to externally validate the prediction model derived from the two remaining cohorts. Third, we compared the performance of our lifestyle model with the 12-item prognostic index developed by Lee and colleagues^8,10^, in the presence and absence of clinical conditions (i.e., diabetes mellitus, cancer, lung disease and heart failure).

The lifestyle prediction model was then translated into a simplified point-based lifestyle index using methods reported previously^29^. Briefly, points were assigned to each risk predictor by dividing the corresponding β coefficient by the β coefficient of a five-year increase in age and rounding to the nearest integer; *with positive values indicating increased risks and negative values indicating decreased risks*. A summary risk score for each participant was the total points for all risk predictors present. The 10-year absolute mortality risk associated with point totals was calculated using flexible parametric proportional hazards model with the pooled baseline survival function. Given no more than 11% participants had missing value, all analyses were conducted among participants with complete measures and adjusted by the individual-level weights provided with the data. All analyses were conducted via Stata SE version 14 (StataCorp, College Station, TX).

### Data availability

The analysis was based on the RAND HRS, the Harmonized ELSA and Harmonized SHARE datasets. These datasets are available from the Gateway to Global Aging Data website: www.g2aging.org.

## Electronic supplementary material

Supplementary Information
