# Supplementary material for: Lifestyle index for mortality prediction using multiple ageing cohorts in the USA, UK and Europe
Source: Sci Rep. 2018 Apr 27;8:6644. doi: 10.1038/s41598-018-24778-1 (PMC5923240; doi:10.1038/s41598-018-24778-1)
Supplement: Supplementary file 1 — Supplementary Information [file 41598_2018_24778_MOESM1_ESM.pdf]

Lifestyle index for mortality prediction using multiple ageing cohorts in the USA, UK and Europe

Jing Liao, Graciela Muniz-Terrera, Shaun Scholes, Yuantao Hao, Yu-ming Chen

Supplementary Figure S1. Baseline survival functions for each cohort (Kaplan-Meier estimates) and the pooled baseline function (S0), estimated from flexible parametric survival model. Data: Health and Retirement Study, English Longitudinal Study of Ageing and Survey of Health, Ageing and Retirement in Europe.

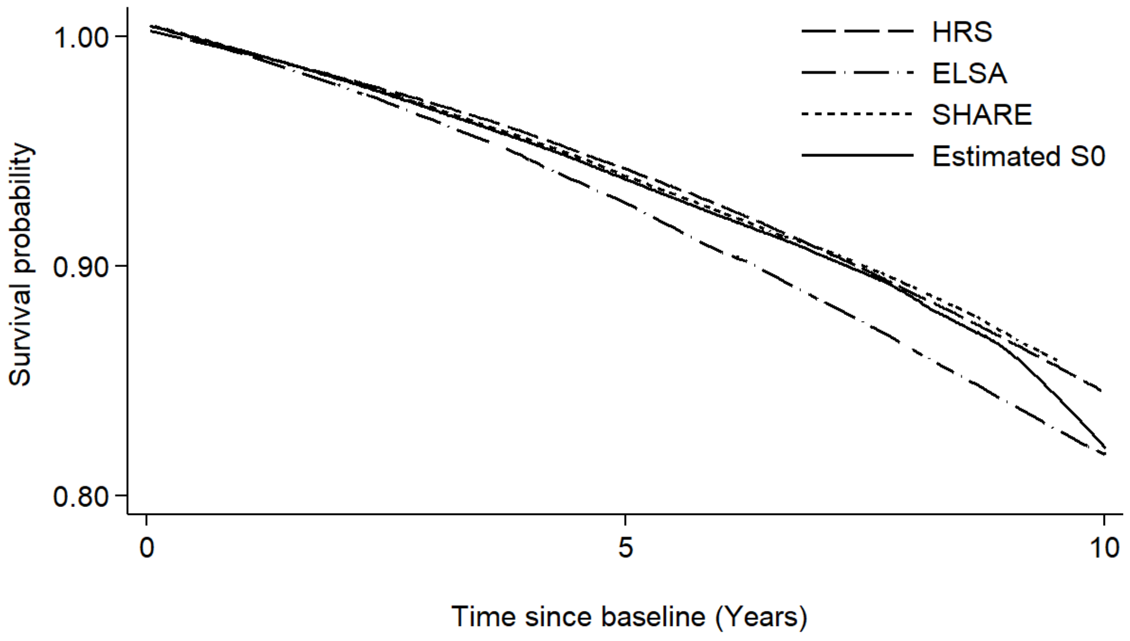

Supplementary Figure S2. Calibration plots for internal-external cross-validation. Smoothed pseudo-values (solid lines) with pointwise 95% confidence interval plotted against predicted event probabilities at 10 years. The dash line is the line of identity, denoting perfect calibration. All three cohorts were interactively used as internal development and external validation cohorts. HRS: Health and Retirement Study; ELSA, English Longitudinal Study of Ageing; SHARE: Survey of Health, Ageing and Retirement in Europe.

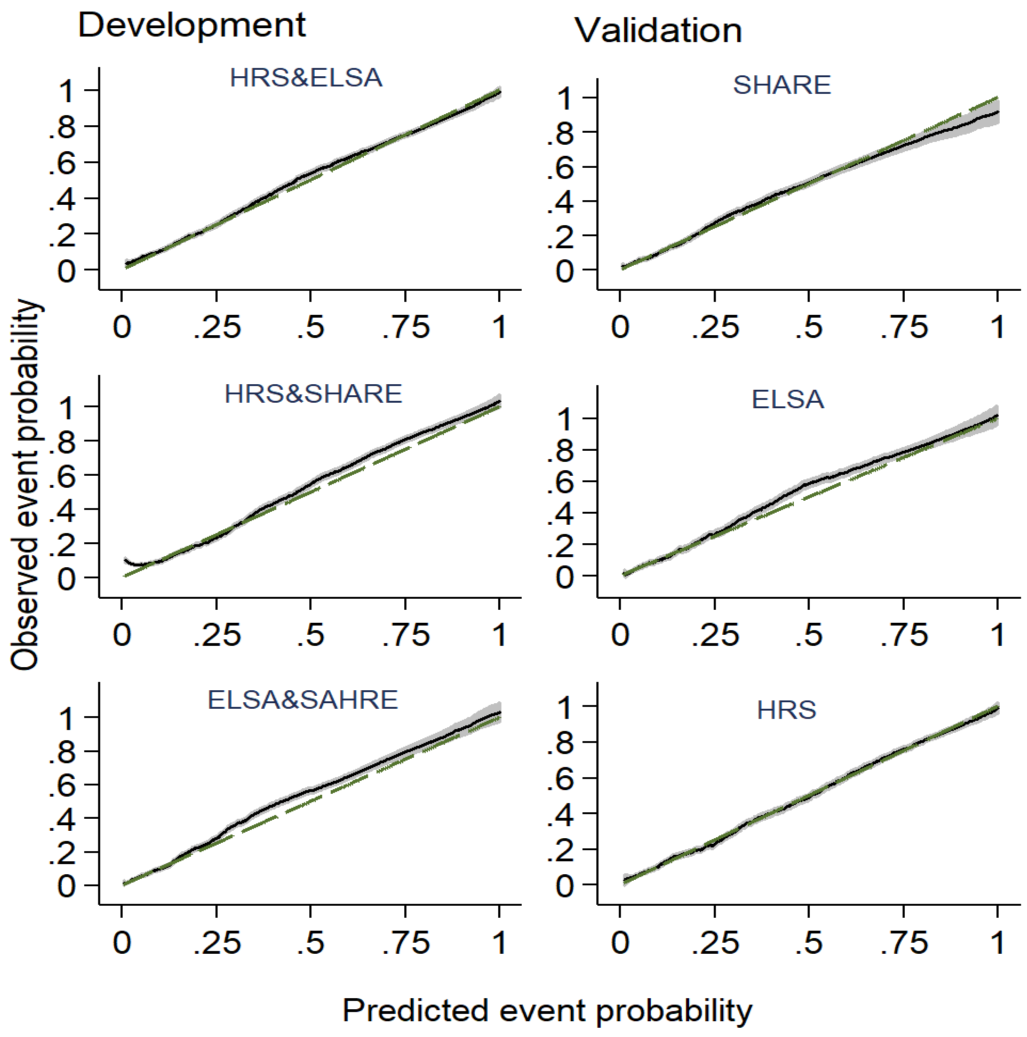

Supplementary Table S1. Baseline lifestyle measures and mortality cases over follow up and cohort [a]

Variables	Overall n=51,668	HRS n=14,879	ELSA n=10,511	SHARE n=26,298
**Age** mean (SD)	65.7（10.2）	67.5 (9.6)	64.6 (10.2)	65.0 (10.5)
**Female** %	55.6	57.6	53.5	54.4
**Smoking status** %				
Never smoker	51.0	41.4	35.9	56.0
Ex-smoker	32.4	44.6	46.2	26.0
Current smoker	16.6	14.0	17.9	18.0
**Drinking status** %				
Nondrinker	38.3	50.8	3.8	31.9
Former drinker	10.8	16.6	7.6	7.7
Moderate drinker	37.9	25.7	84.3	44.3
Heavy drinker	13.0	6.9	4.3	16.2
**Physical activity level** %				
High	40.1	28.5	28.2	46.1
Medium	44.5	50.3	54.2	41.5
Low	15.4	21.3	17.6	12.4
**Sleep quality (restless)** %				
No	68.6	72.0	59.6	66.9
Yes	31.4	28.0	40.4	33.1
**BMI** (kg/m2) %				
≤20	4.3	5.0	2.4	3.9
>20-25	34.7	31.4	26.2	36.5
>25-30	41.1	38.7	45.0	42.4
>30	19.9	24.9	26.4	17.2
**Total Deaths** N (%)	10,240 (19.8)	5,461 (36.7)	2,091 (19.9)	2,688 (10.2)

HRS, Health and Retirement Study; ELSA, English Longitudinal Study of Ageing; SHARE, Survey of Health, Ageing and Retirement in Europe; SD, standard deviation; BMI, body mass index.

[a.] Variables were weighted by the individual-level weights.

Supplementary Text S1. Survey-specific lifestyle factor harmonizing strategy

1）Smoking

All surveys asked about past and current smoking. Despite similar survey items used, it is to be noted that while HRS and ELSA asked participants whether they ever smoked cigarettes, SHARE instead asked whether they smoked cigarettes or other types of tobacco for at least one year. These variations may result in underestimation of overall tobacco use in HRS and ELSA, and lower past smoking behaviour in SHARE. Based on these two items, a three-category smoking status variable (i.e., never smoker=0, ex-smoker=1, or current smoker=2) was derived.

2）Drinking

Drinking status was derived from questions on frequency and quantity of alcohol consumption. All surveys asked whether respondents have ever drunk any alcoholic drinks but vary in timespan. HRS quantified daily drinks consumed over the last three months, while SHARE asked about drinking frequency, particularly occasions with two more drinks per day in the last six months. ELSA assessed drinking frequency over the past 12 months, with more detailed information on total units of weekly alcohol consumption collected in the Health Survey for England (ELSA Wave 0). Given the majority of the respondents (82.5%) did not change their drinking habits between ELSA Wave 1 and the Health Survey for England (HSE), more detailed information on total units of alcohol consumed per week over last the 12 months from HSE interviews was also used to identify former drinker.

In light of the literature[1,2] and measures available, we classed drinking status into the following four groups: 1) nondrinker (=0) reporting zero alcohol consumption; 2) former drinker (=1) who reported drinking before but quit drinking now; 3) moderate drinker (=2, reference) ≦2 drinks/day (HRS and SHARE) or less than twice/day (ELSA), 4) while beyond these boundaries participants were grouped as heavy drinkers (=3).

3）Physical activity

Questions on vigorous and moderate exercise are comparable across surveys. In HRS analysis baseline (Wave 6), only one question regarding vigorous physical activity was asked: ‘On average over the last 12 months have you participated in vigorous physical activity or exercise three times a week or more?’ (Answer: Yes, No). Thus, this item in combination with HRS Wave 7’s questions on both vigorous and moderate exercise were used to define the physical activity level of HRS participants. We define three levels of physical activity, which are high (=0, vigorous exercise at least once per week), medium (=1, moderate exercise at least once per week, reference), and low (=2, hardly ever/never moderate-vigorous exercise). There were less than 10% of participants who took either moderate or vigorous exercise 1–3 times a month in each survey. Given participants belonging to this group had no statistically significant differences in mortality rate in comparison with the reference group. These participants were grouped into the medium level physical activity category.

4) Sleep quality

A comparable question on sleep quality (restless) was asked across surveys as part of the mental health assessment, with binary answer no (=0) and yes (=1).

5) BMI

Body mass index (BMI) was measured consistently across the surveys: weight (kg) divided by height (in meters) squared (m^2). We stratified the continuous BMI into four classes ≤20, >20-25, >25-30 and>30 kg/ m^2 as recommended[3]: where the 25-30 kg/m^2 category was chosen as the reference category given the BMI-mortality U-shaped curve with the nadir between 24.0-30.9 kg/m^2. [4]

References:

1. Lang I, Guralnik J, Wallace RB, Melzer D. What level of alcohol consumption is hazardous for older people? Functioning and mortality in U.S. and English national cohorts. Journal of the American Geriatrics Society. 2007;55(1):49-57.
2. Stockwell T, Zhao J, Panwar S, Roemer A, Naimi T, Chikritzhs T. Do "Moderate" Drinkers Have Reduced Mortality Risk? A Systematic Review and Meta-Analysis of Alcohol Consumption and All-Cause Mortality. Journal of Studies on Alcohol and Drugs. 2016;77(2):185-98.
3. Donini LM, Savina C, Gennaro E, De Felice M, Rosano A, Pandolfo M, et al. A systematic review of the literature concerning the relationship between obesity and mortality in the elderly. The Journal of Nutrition, Health & Aging. 2012;16(1):89-98.
4. Winter JE, MacInnis RJ, Wattanapenpaiboon N, Nowson CA. BMI and all-cause mortality in older adults: a meta-analysis. The American Journal of Clinical Nutrition. 2014;99(4):875-90.
